# Forecasting COVID-19 Confirmed Cases Using Empirical Data Analysis in Korea

**DOI:** 10.3390/healthcare9030254

**Published:** 2021-03-01

**Authors:** Da Hye Lee, Youn Su Kim, Young Youp Koh, Kwang Yoon Song, In Hong Chang

**Affiliations:** 1 Department of Computer Science and Statistics, Chosun University, Gwangju, 61452, Korea; is_hye@chosun.kr (D.H.L.); imk92315@naver.com (Y.S.K.); 2 Department of Internal Medicine, College of Medicine and Medical School, Chosun University, Gwangju 61452, Korea; yykoh@chosun.ac.kr

**Keywords:** time-series, ARIMA, forecasting, confirmed cases, COVID-19, pandemic

## Abstract

From November to December 2020, the third wave of COVID-19 cases in Korea is ongoing. The government increased Seoul’s social distancing to the 2.5 level, and the number of confirmed cases is increasing daily. Due to a shortage of hospital beds, treatment is difficult. Furthermore, gatherings at the end of the year and the beginning of next year are expected to worsen the effects. The purpose of this paper is to emphasize the importance of prediction timing rather than prediction of the number of confirmed cases. Thus, in this study, five groups were set according to minimum, maximum, and high variability. Through empirical data analysis, the groups were subdivided into a total of 19 cases. The cumulative number of COVID-19 confirmed cases is predicted using the auto regressive integrated moving average (ARIMA) model and compared with the actual number of confirmed cases. Through group and case-by-case prediction, forecasts can accurately determine decreasing and increasing trends. To prevent further spread of COVID-19, urgent and strong government restrictions are needed. This study will help the government and the Korea Disease Control and Prevention Agency (KDCA) to respond systematically to a future surge in confirmed cases.

## 1. Introduction

The COVID-19 pandemic has had a significant impact on human life. The G20 Summit held in a virtual conference on March 2020 to discuss pending global issues resulting from COVID-19. Coping and confronting the pandemic includes activities such as protecting lives, protecting jobs and income, restoring trust, preserving financial stability, restoring growth, minimizing disruption of trade and global supply chains, and providing assistance to countries in need of support. COVID-19 has caused major economic losses, paralyzing national economies around the world. The International Monetary Fund (IMF) predicted that global trade volume would shrink by 10.4% on-year [1]. The World Bank Group (WBG) is expecting that the global trade volume will drop 5.2% and to have its worst year since World War II [2].

COVID-19 has been called a novel coronavirus (2019-nCoV), but on 11 March 2020, the World Health Organization (WHO) announced its official name as COVID-19 [3]. On 13 February 2020, the International Committee on Taxonomy of Viruses (ICTV) officially announced the virus’ name as SARS-CoV-2. Coronavirus is a ribonucleic acid (RNA) virus that causes respiratory diseases, such as colds. It was named coronavirus because its outer skin is shaped like a crown surrounded by bumps. It causes infection in a variety of animals, including humans. The WHO classifies pandemic alarm levels from 1 to 6, according to the infectious disease risk. This pandemic corresponds to the highest warning level—6. When an infectious disease spreads worldwide and spreads across continents, it is called a pandemic. Thus far, the WHO has declared three pandemics: the Hong Kong Flu in 1968, the Swine Flu in 2009, and COVID-19 in 2020 [4].

Until recently, the top five affected countries were as follows: the United States death toll record with 17 million, India with 10 million, Brazil with 7 million, Russia with 2.7 million, and France with 2.4 million. In terms of death rate, Mexico has the highest death rate at 9.1%, China has 5.3%, Iran has 4.7%, and Italy has 3.5%. In Korea, the cumulative number of confirmed cases is about 47,000, and the death rate is approximately 1.4% [5].

Various studies have been conducted on past pandemic infections and disease. Guan et al. [6] predicted the incidence of hepatitis A virus (HAV) using an auto regressive integrated moving average (ARIMA) model and an artificial neural network (ANN). Earnest et al. [7] forecasted the number of confirmed cases by applying the ARIMA model to the number of confirmed cases per day for severe acute respiratory syndrome (SARS). By applying ARIMA to China’s HFRS data, Liu et al. [8] predicted the incidence of hemorrhagic fever with renal syndrome (HFRS) from 2009 to 2011. Wu et al. [9] predicted the incidence of HFRS over one year by using a hybrid model that combines ARIMA, a generalized regression neural network (GRNN), and the non-linear autoregressive neural network (NARNN) with ARIMA. Nsoesie et al. [10] tried to predict the hantavirus pulmonary syndrome (HPS) using an ARIMA model. Chen et al. [11] used the seasonal autoregressive integrated moving average (SARIMA) to predict the incidence of influenza in China; they found that the incidence rate varies according to region and season.

Based on past infectious diseases, research related to COVID-19 has also been actively conducted. Using a differential equation model that reflected social distancing and transmission rate as parameters, Webb et al. [12] predicted and compared the number of confirmed cases considering the number of report and the presence of symptoms in Italy, Spain, and Korea. This demonstrates the importance of controlling COVID-19 infection through social distancing. Alakus et al. [13] developed a prediction algorithm using deep learning and had a positive impact on clinical prediction studies of COVID-19. Pham [14] studied the cumulative number of deaths, the mortality per capita per unit time, and the maximum total number of deaths as functions, and the solution of differential equations composed of the functions is proposed as the numerical model of COVID-19. Pham [15] generalized by introducing a function of recovered cases to the model in [14]. Additionally, Pham [16] developed a new mathematical model by introducing the time-dependent effort of social restrictions—the resumption of states, wearing masks, and social distancing. Arias et al. [17] suggested a generalized logistics regression to predict the number of cases of COVID-19.

In addition to the aforementioned methods, studies have also been conducted using the ARIMA model to estimate the spread of COVID-19, examples of which are as follows. Using ARIMA and Richard’s model, Kumar et al. [18] conducted a study that forecast the population impact of COVID-19 in India compare goodness-of-fit for models. Petropoulos et al. [19] predicted the number of COVID-19 patients in a short period of time using a simple time series in Denmark, Norway, and Sweden. Additionally, [19] tracked and compared the stringency level of each country. Using the ARIMA model, Ceylan [20] predicted the number of COVID-19 cases in Italy, Spain, and France. Alzahrani et al. [21] forecasted the number of COVID-19 confirmed cases in Saudi Arabia for the next four weeks. Yang et al. [22] predicted the number of cases in Italy for the next few days. Kufel [23] presented ARIMA to forecast the rate of infection in 32 European countries over the next seven days. In addition, there is a variety of research that studies the impact of COVID-19 [24,25,26,27,28,29,30].

In this paper, we apply the ARIMA model and empirical data analysis to forecast the number of confirmed COVID-19 cases in Korea. Using actual data, dividing the wave into several cases, predicting the number of cumulative confirmed cases for each case, and comparing the criteria. In doing so, we emphasize the importance of timing of forecasting to make a meaningful forecast. In particular, the period from 20 January 2020 (first confirmed case) to 26 October 2020 (the beginning of the third wave of COVID-19) is divided into five groups, which are subdivided into a total of 19 cases (the division is detailed in Section 2). Section 2 briefly describes the material and methods. Additionally, the current status of confirmed cases in Korea, empirical data analysis of group and case information, ARIMA models, and criteria are introduced. Section 3 presents the analysis and results. Section 4 concludes the paper.

## 2. Material and Methods

### 2.1. The Number of COVID-19 Confirmed Cases in Korea

**Figure 1:**
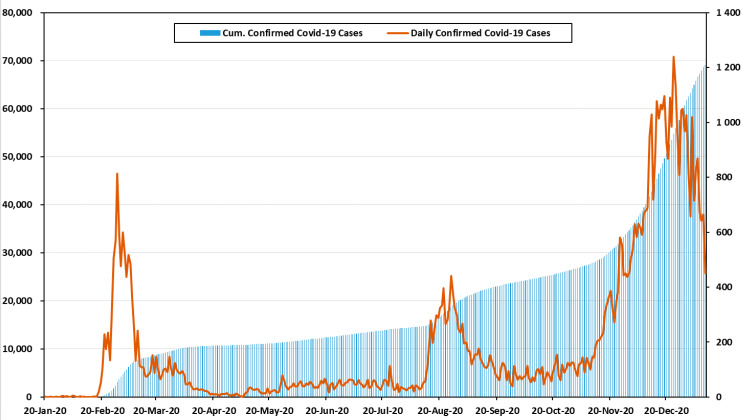
The number of confirmed cases and cumulative confirmed cases of COVID-19 in Korea in 2020 (including imported cases).

Figure 1 shows the number of confirmed cases and cumulative confirmed cases by month in Korea [31]. On 20 January 2020, a tourist from Wuhan became the first confirmed case in Korea. Then, 11 cases were reported, bringing the cumulative number of confirmed cases to 12. In February and March, the number of confirmed cases increased sharply. The primary cause of infections was indoor religious gatherings. Within three months of the first outbreak, the cumulative number of confirmed cases reached 9887. The period between February and April 2020 is defined as the first wave of COVID-19 in Korea [31,32].

After the first wave, the number of confirmed cases decreased rapidly and there was a stable infection rate across the country. Nevertheless, in August and September, the second wave was generated by political rallies and church gatherings. During the second wave, the cases increased sharply, and the government raised social distancing to level 2. There were 2757 cases in October, which was only slightly lower than in September. This period showed a stable infection rate, in comparison to other waves, but it included the day with the largest increase in confirmed cases; this study did not thoroughly address the third wave, because it is still underway [31,33].

From November to present, the number of confirmed cases increased rapidly again. This is defined as the third wave. In November, the total number of cases was 8017. Small gatherings among families and friends accounted for more than 20% of the third wave’s infections. Some of the provincial governments decided to raise the social distancing level to 2.5, which is the second highest. Worst of all, the confirmed cases in Seoul are being housed in retrofitted containers because of hospital bed shortages. The government and citizens fear the need to raise social distancing to level 3 [31,34].

All information related to confirmed cases in this paper was provided by the government and was aggregated daily at midnight (00:00) [31].

### 2.2. Information of Groups and Cases Using Empirical Data Analysis

#### 2.2.1. Empirical Data Analysis

Empirical analysis is an evidence-based approach to the study and interpretation of information. The empirical approach relies on real-world data, metrics, and results, rather than theories and concepts. Empirical analysis is a common approach used to study probable answers through quantified observations of empirical evidence. However, empirical analysis never gives an absolute answer, only the most likely answer based on probability.

We can formulate the increasing number of confirmed cases of COVID-19 as follows:

(1) $$y^{\prime }\left(t\right)=\underset{∆t\to 0}{\lim}\frac{y\left(t+∆t\right)-y\left(t\right)}{∆t}$$

where $y^{\prime }\left(t\right)$ illustrates the increasing number of confirmed cases of COVID-19 during the time interval ∆t. Then, y(t) is the observed cumulative number of confirmed cases of COVID-19 over time t. Therefore, y(t+∆t) denotes the observed cumulative number of confirmed cases of COVID-19 over time t+∆t. Given different values of ∆t, we are interested in investigating the pattern of $y^{\prime }\left(t\right)$.

#### 2.2.2. Information of Groups and Cases

**Figure 2:**
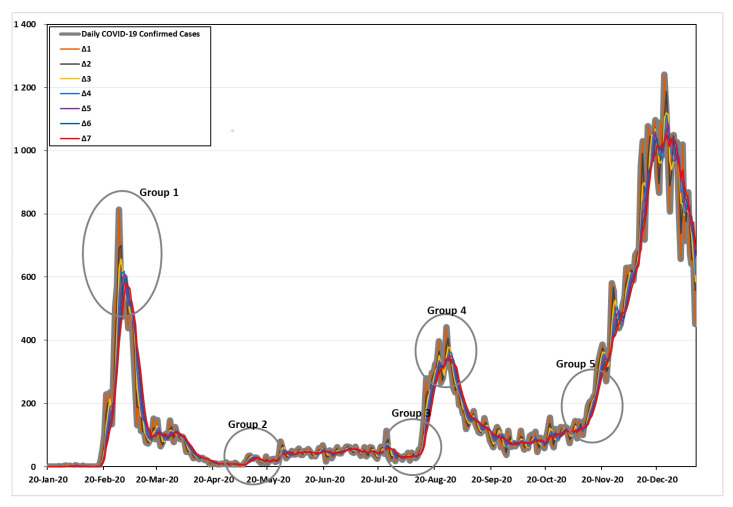
The increasing number of confirmed cases of COVID-19 by time interval.

Figure 2 shows the increasing number of confirmed cases of COVID-19 during the time interval ∆t. As shown in Figure 2, the five points of high variability were divided and examined in detail. The criteria for defining the five groups are as follows: Group 1 and Group 4 were based on the day when the number of confirmed cases per day was the highest in the first and second waves. Group 2 was based on the day when the number of confirmed cases was the lowest. Last, Group 3 and Group 5 were based on the days with the greatest variability (the point at which more than 100 confirmed cases began to appear), which signaled the beginning of the second and third waves.

Details can be found in Table 1. In Group 1, with the time interval ∆t = 1, the maximum frequency was 813 cases (28 February 2020), the time intervals ∆t = 2–4 were 699.5, 656.7, and 618.8 cases (29 February 2020), and the time internals ∆t = 4–5 were 618.8 and 609.2 cases (2 March 2020). Time internals ∆t = 6–7 were 593.7 and 581.0 cases (3 March 2020) in the first wave of the COVID-19 pandemic, respectively.

In Group 2, with the time interval ∆t = 1, 2, and 7, the minimum frequencies were 2, 2.5, and 6.4 cases (5 May 2020). The time intervals ∆t = 3–4 and 6–7 were 3, 4.3, 6, and 6.4 cases (6 May 2020), and the time intervals ∆t = 5 was 5.8 cases (7 May 2020), respectively.

In Group 3, with the time interval ∆t = 1, the frequency with high variability (based on more than 100 cases) was 103 cases (13 August 2020). The time intervals ∆t = 2–3 were 134.5 and 108.3 cases (14 August 2020). The time internals ∆t = 4–7 were 151, 131.6, 115.3, and 102.9 cases (15 August 2020) before the second wave of the COVID-19 pandemic, respectively.

In Group 4, with the time interval ∆t = 1, the maximum frequency was 441 cases (26 August 2020). The time intervals ∆t = 2 and 6–7 were 406, 345.8, and 343.9 cases (27 August 2020). The time internals ∆t = 3–4 were 378.3 and 363.8 cases (28 August 2020). Time internals ∆t = 5 was 350.8 cases (29 August 2020) in the second wave of the COVID-19 pandemic.

In Group 5, with the time interval ∆t = 1–2, the frequency with high variability (based on more than 100 cases) was 119 and 105 cases (21 October 2020). The time intervals ∆t = 3–4 were 121.7 and 105.8 cases (22 October 2020). The time internals ∆t = 5 was 100 cases (23 October 2020); the time internals ∆t = 6 was 103.7 cases (25 October 2020); and the time internals ∆t = 7 was 101.4 cases (26 October 2020) before the third wave of the COVID-19 pandemic, respectively.

As shown in Table 2, we set cases by date for forecast analysis based on the time point mentioned in each group. In addition, it was used for predictive analysis using the data up to the mentioned time point.

### 2.3. Time Series

In the autoregressive (AR) model, the partial autocorrelation coefficient (PAC) had a significant spike, and the autocorrelation coefficient (AC) decreased in sequence. In this case, the order of AR (p) is determined based on the number of significant spikes of the PAC. The formula for the AR (p) model is as follows:

(2) $$Y_{t}=\epsilon_{t}+\alpha_{1}Y_{t-1}+\alpha_{2}Y_{t-2}+\cdots \alpha_{p}Y_{t-p}$$

Unlike AR, in the moving average (MA) model, the AC has a significant spike. The PAC decreases in sequence, and the order q of the MA model is determined based on the number of significant spikes of the AC. The formula for the MA (q) model is as follows:

(3) $$Y_{t}=\epsilon_{t}-\beta_{1}\epsilon_{t-1}-\beta_{2}\epsilon_{t-2}-\cdots \beta_{q}\epsilon_{t-q}$$

The autoregressive moving average (ARMA) model shows a form of sequentially decreasing in both the AC and the PAC. The formula is as follows:

(4) $$Y_{t}=\alpha_{1}Y_{t-1}+\alpha_{2}Y_{t-2}+\cdots \alpha_{p}Y_{t-p}+\epsilon_{t}-\beta_{1}\epsilon_{t-1}-\beta_{2}\epsilon_{t-2}-\cdots \beta_{q}\epsilon_{t-q}$$

where $\epsilon_{t}$ is called the error or white noise. The $\epsilon_{t}$ is assumed to be independently normal distribution. The ARIMA model converts a non-stationary time series data into a stationary time series that is expressed as ARIMA (p,d,q), where p is the order of the AR model, d is the differencing order, and q is the order of the MA model. For example, AR (1) is equivalent to ARIMA (1,0,0), and MA (2) is equivalent to ARIMA (0,0,2).

There is no clear trend in the stationary time series, and the average and variance are constant over time. In the case of a known time series analysis model, analysis is possible when the data is in the form of time series data that shows normality without trend or seasonality. In the case of data having a long period, a trend with a sudden and unpredictable change in direction, or data showing seasonality, the analysis is conducted after making the data in the form of a stationary time series through the difference using the difference between observed values. To check whether it is a normal time series or a non-stationary time series, check through a sequence chart or ACF (auto correlation function) [35].

This paper dealt only with the ARIMA (p,2,q) model. In general, a non-stationary time series becomes a stationary time series by a first or second differencing. In the data of this study, when the difference was 0 or 1, the sequence chart had an inconsistent form of mean and variance, and it can be seen that the ACF had an abnormal time series in the form of slowly decreasing. When the difference was 2, the mean and variance appeared in a certain form, indicating that the time series was normal.

When d=1, the cumulative number of confirmed cases predicted by the ARIMA model, gradually decreased or showed a negative value, which is a contradiction. However, when d=2, the predicted value of the cumulative cases increased stably, so the ARIMA (p,2,q) model was used.

### 2.4. Criteria for the Comparion of Goodness-of-Fit

To compare the goodness-of-fit by ARIMA for each case, the following four criteria were used:

First, root mean square error (RMSE) is as follows:

(5) $$\mathrm{RMSE}=\sqrt{\frac{1}{n}\overset{n}{\underset{t=1}{\sum }}e_{t}{}^{2}}$$

Second, mean absolute error (MAE) is as follows:

(6) $$\mathrm{MAE}=\frac{1}{n}\overset{n}{\underset{t=1}{\sum }}|e_{t}{}^{2}|$$

Third, mean absolute percentage error (MAPE) is as follows:

(7) $$\mathrm{MAPE}=\frac{100}{n}\overset{n}{\underset{t=1}{\sum }}e_{t}{}^{2}$$

Finally, the sum of square error (SSE) is as follows:

(8) $$\mathrm{SSE}=\overset{\left(n+1\right)+14}{\underset{t=n+1}{\sum }}{\left(Y_{t}-\hat{Y_{t}}\right)}^{2}$$

Here, $e_{t}$ is the difference (error) between the actual cumulative number of cases $Y_{t}$ and the predicted value $\hat{Y_{t}}$ of the ARIMA model at time t. Additionally, n is the length of time t. The SSE was calculated as the difference between the predicted values and the data for 14 days—two weeks from the end of the truncated case. The smaller the values of all four criteria mentioned above, the better the fit, relative to other models.

## 3. Results

For the data set, the time series method was applied to compare the criteria of each section using SPSS 25 (IBM, Armonk, NY, USA). The ARIMA (p,d,q) models were fitted p = 0, 1, …, 5, d = 2, q = 0, 1, …, 5 for 19 cases, with 684 models to be compared. Among them, only the top six models of each case were selected based on the RMSE.

### 3.1. Prediction of Cumulative Confirmed Cases of COVID-19 by Group and Case Using ARIMA

#### 3.1.1. Comparison of Goodness-of-Fit by Group and Case

Table 3, Table 4, Table 5, Table 6 and Table 7 show the fitting ARIMA models and criteria for groups and cases, and sorts by RMSE (in ascending order).

As can be seen in Table 3, in case 1, the RMSE of ARIMA (5,2,5) was 41.181, which was closer to the actual data than other models. In addition, the MAE of the model was 21.819, which was the smallest of all models. The MAPE of ARIMA (3,2,3) was 170.642, which was the smallest among case 1. In case 2, the RMSE and MAE of ARIMA (5,2,5) were the smallest. Based on MAPE, the value of ARIMA (4,2,2) was the closest to the actual data. In Cases 3 and 4, all criteria of ARIMA (5,2,5) appeared to be predictive models with the best descriptive.

As can be seen in Table 4, in case 5, the RMSE of ARIMA (2,2,5) was 56.172, which was the smallest among case 5. Based on MAPE, the value of ARIMA (1,2,5) was 5.741, which was the smallest. The MAE of ARIMA (5,2,5) was 27.800, which was the smallest. In case 6, based on the RMSE, the value of ARIMA (2,2,5) was 55.895, which was the smallest. The MAPE of ARIMA (3,2,5) was 5.668, which appeared to be a predictive model with the best descriptive. The MAE of ARIMA (5,2,5) was 27.637, which was the smallest. In case 7, the RMSE and MAPE of ARIMA (2,2,5) were the closest among case 7, and the MAE of ARIMA (5,2,5) was the smallest of all the models.

As can be seen in Table 5, in case 8, the RMSE and MAE of ARIMA (2,2,5) were the closest among case 8. The MAPE of ARIMA (3,2,5) was 3.324, which was the smallest among the other models. In case 9, the RMSE of ARIMA (2,2,5), the MAPE of ARIMA (4,2,5), and the MAE of ARIMA (3,2,5) were 41.912, 3.658, and 21.489, which were the closest to the actual data in comparison to the other models. In case 10, the RMSE of ARIMA (2,2,5) was 42.796, which was the closest to the others. The MAPE of ARIMA (2,2,3) and the MAE of ARIMA (5,2,4) appeared to be predictive models with the best goodness-of-fit.

As can be seen in Table 6, in case 11, the RMSE of ARIMA (5,2,5) was 44.253, which was closer to the actual data than the other models. Based on the MAPE and MAE, the values of ARIMA (3,2,5) were the closest among case 11. In case 12, the RMSE of ARIMA (3,2,5) was 44.405, which appeared to be the best predictive value. The MAPE of ARIMA (1,2,5) was 4.467, which was the smallest. The MAE of ARIMA (4,2,5) was 23.207, which was the closest to the others. In cases 13 and 14, the RMSE and MAPE of ARIMA (3,2,5) provided the best fit. Based on MAE, ARIMA (4,2,4) appeared to be a predictive model with the best fit.

As can be seen in Table 7, in case 15, the RMSE and MAE of ARIMA (3,2,5) provided the best fit. The MAPE of ARIMA (2,2,5) was 2.495, which was closer to the actual data than the other models. In case 16, the RMSE and MAE of ARIMA (4,2,5) provided the best fit. The MAPE of ARIMA (4,2,4) was 2.609, which predicted significantly better results than the others. In case 17, as in case 15, the RMSE and MAE of ARIMA (3,2,5) show the best fit. The MAPE of ARIMA (1,2,5) was the smallest. In case 18, all criteria of ARIMA (3,2,5) provided the best fit among the other models. In case 19, as in case 15, the RMSE and MAE of ARIMA (3,2,5) were predictive with the best fit. The MAPE of ARIMA (5,2,2) was 2.386, which was the closest to the actual data.

#### 3.1.2. Comparison of Predictive Value by Group and Case

Table 8 describes the results of the ARIMA models for each group and case, based on SSE. Here, note means the time interval, including the variability (maximum, minimum, and high variability of the point at which more than 100 confirmed cases began to appear), elapsed from the base date of each group.

As can be seen in Table 8, in Group 1, the SSE of ARIMA (4,2,5) for case 2 was 138,245,907, which was significantly smaller than the others. In Group 2, the SSE of ARIMA (5,2,5) for case 7 was 21,750, which was the smallest. The SSE of ARIMA (1,2,5) for case 10 in Group 3, ARIMA (4,2,5) for case 14 in Group 4, and ARIMA (2,2,5) for case 17 in Group 5 were the closest to actual data compared to the other models in the same group. We confirmed that the analysis should be performed taking into account the time interval of the last five days or more, including the maximum, minimum, and high variability (when more than 100 confirmed cases started to appear).

For reference, it was confirmed that the analysis should be performed taking into account the time interval of the last five days or more, including the maximum, minimum, and high degeneration (when more than 100 confirmed cases started to appear).

Note the consideration of the maximum, minimum, and expensive modification, (a confirmed case is the time more than 100 people begin to appear) over the last five days, confirmed that this analysis should be done.

Based on the note above, ∆t of the best model in Group 1 was 2, 3, and 4, a period that was the initial period of the COVID-19 outbreak. Thus, its data was small; ∆t was smaller than other groups. In Groups 2, 4, and 5, the values of the best models for each group were 5. In Group 3, ∆t of the best model was 4, 5, 6, and 7 and the minimum was 4. That is, we found that the best prediction in Group 3 was to analyze it using the data up to the point of high variability (minimum and maximum) over four days. Except for Group 1, which was unstable due to low data, the remaining groups were required to predict using the data up to the point of high variability (minimum and maximum) for the last five days.

### 3.2. Results of Fitting and Forecasting for the Latest Period Using ARIMA

The ARIMA model was fitted to the data set of confirmed COVID-19 cases, including the data set from the latest period of the third wave outbreak (up to 27 December 2020). As in Section 3.1.1, ARIMA (p,d,q) models were fitted p = 0, 1, …, 5, d = 2, q = 0, 1, …, 5 for 19 cases. Table 9 lists the top 10 based on the RMSE among the fitted ARIMA models.

Based on the RMSE, ARIMA (3,2,5) provides the best fit, the value was 53.031. Additionally, the MAE of the model was 29.780, the closest to actual model than others. Compared to other models based on MAPE, the value of ARIMA (1,2,5) was 3.860, appeared to be the best predictive model. The model with the least SSE in each group in Table 8 also had smaller RMSE, MAPE, and MAE values compared to cases in the same group. Therefore, we estimated the predicted values and 95% confidence intervals over the next 14 days for the best models, ARIMA (3,2,5) and ARIMA (1,2,5) based on three criteria.

**Figure 3:**
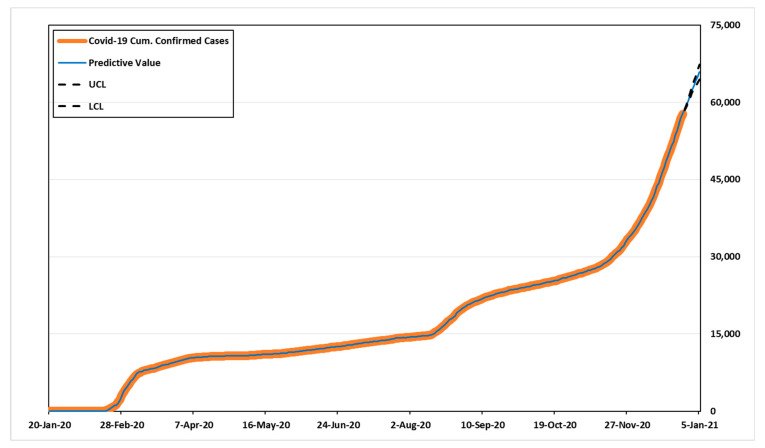
Time-series plot for ARIMA (3,2,5).

**Figure 4:**
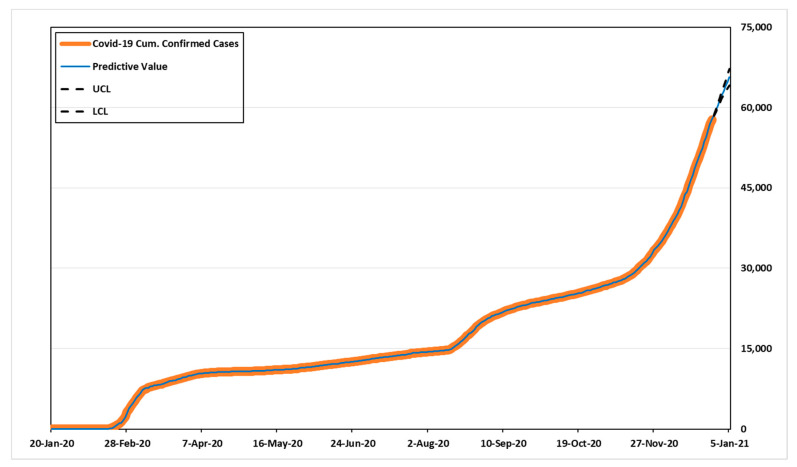
Time-series plot for the best ARIMA (1,2,5).

Table 10 shows the predicted values, UCL (upper confidence limit), and LCL (lower confidence limit). According to Table 10, the number of cumulative confirmed cases for the next 14 days might be 58,532–70,389 in ARIMA (3,2,5), and 58,533–69,877 in ARIMA (1,2,5). Figure 3 and Figure 4 show the predicted values, 95% confidence intervals, and actual data values for each model.

## 4. Discussion

In Section 3, we used ARIMA to compare the criteria of each case using data sets from Korea. The period between 20 January to 26 October 2020 was divided into five based on (1) peak of the first wave; (2) the day when the increase in confirmed cases is at its minimum; (3) the day when the variability of the confirmed case is high before the peak of the second wave; (4) peak of the second wave; and (5) the day when the variability of the confirmed cases is high before the peak of the third wave. Table 3, Table 4, Table 5, Table 6 and Table 7 show the top six results by comparing the goodness-of-fit of the ARIMA model for each group and case, and Table 8 shows the top five results based on SSE to examine the predicted values.

In general, if the goodness-of-fit is high, the predicted value is thought to be high, but the results were different. As can be seen from the note of the results in Table 8, the SSE value of the ARIMA model derived using ∆t 5 was significantly lower than that of other models.

It is recommended because it performs much better at predicting the number of confirmed cases using data at each point in time of the time interval 5, i.e., the average data of 5 days. By predicting the number of confirmed patients based on the results of analysis at various points in time using empirical data analysis and the ARIMA model using it, it is possible to preemptively respond to the variability (increase, decrease, rapid increase, etc.) of the number of confirmed patients through daily updates.

Additionally, in Korea, since the case definition is clear and data collection is almost in real time, the predictive power of the ARIMA model is relatively excellent and stable. There were unpredictable events due to the blind spot, but the blind spot is expected to gradually decrease due to the learning effect and preemptive examination on the similar exposure pathway. In addition, they successfully conducted a blind test as a way to cope with the phenomenon of avoiding tests due to social stigma, and there is a foundation for imposing legal sanctions in case of false reports on the route of infection. Prediction through the ARIMA model provides an important basis for KDCA to predict the necessary severe disease constant and prepare it in advance. In Korea, the proportion of public medical services is small, so the number of beds that can treat critically ill patients is limited. This is because it takes time to secure the number of severe illnesses by seeking cooperation from the private medical field. The accuracy of the prediction model is expected to improve as data is accumulated. However, there is a need for a model that can reflect the effects of external factors such as the effect of policy measures such as adjustment of the quarantine stage and the influx of mutant viruses.

## 5. Conclusions

This study aimed to suggest an appropriate prediction time point to significantly predict the number of confirmed cases. To significantly predict the number of confirmed COVID-19 cases in Korea, we proposed it should be analyzed and predicted using data at each point in time of the time interval 5, i.e., the average data of 5 days. Forecasting at this time can clearly confirm whether the number of cases will increase or decrease in the future.

The ARIMA model was fitted using the most recent data in progress for the third wave. As a result of predicting the number of cumulative confirmed cases for the next 14 days based on the best models of each criterion, the number of cumulative confirmed cases by the beginning of next year was expected to reach 70,000. Currently, Korea has a shortage of hospital beds. The results are expected to effectively estimate at the point the number of beds required by predicting variability (decrease and, increase) and the number of confirmed cases. In addition, this study is expected to help the government and Korea Disease Control and Prevention Agency (KDCA) to respond systematically to a future surge in confirmed cases.

However, it is difficult to accurately predict the changing cases, because various factors affect the increase in the number of confirmed cases. Furthermore, the influence of mass inflection is large. Therefore, it is necessary to study various techniques, such as reinforcement of machine learning, modeling research based on deep learning, and the application of prediction algorithms.

## Figures and Tables

**Table 1:** The number of confirmed cases of COVID-19 during time interval ∆t by Group.

**Group**	**Date**	Number of Confirmed Cases of COVID-19		Number of Confirmed Cases of COVID-19during Time Interval ∆t						
		Daily	Cum.	∆t=1	∆t=2	∆t=3	∆t=4	∆t=5	∆t=6	∆t=7
Group 1	27 February 2020	571	2337	571.0	538.0	453.3	373.5	345.8	317.3	304.7
	28 February 2020	813	3150	813.0	692.0	629.7	543.3	461.4	423.7	388.1
	29 February 2020	586	3736	586.0	699.5	656.7	618.8	551.8	482.2	446.9
	1 March 2020	476	4212	476.0	531.0	625.0	611.5	590.2	539.2	481.3
	2 March 2020	600	4812	600.0	538.0	554.0	618.8	609.2	591.8	547.9
	3 March 2020	516	5328	516.0	558.0	530.7	544.5	598.2	593.7	581.0
	4 March 2020	438	5766	438.0	477.0	518.0	507.5	523.2	571.5	571.4
Group 2	4 May 2020	3	10,804	3.0	5.5	8.0	7.5	7.8	7.2	7.4
	5 May 2020	2	10,806	2.0	2.5	4.3	6.5	6.4	6.8	6.4
	6 May 2020	4	10,810	4.0	3.0	3.0	4.3	6.0	6.0	6.4
	7 May 2020	12	10,822	12.0	8.0	6.0	5.3	5.8	7.0	6.9
	8 May 2020	18	10,840	18.0	15.0	11.3	9.0	7.8	7.8	8.6
Group 3	12 August 2020	56	14,770	56.0	55.0	48.0	43.0	41.6	41.8	38.7
	13 August 2020	103	14,873	103.0	79.5	71.0	61.8	55.0	51.8	50.6
	14 August 2020	166	15,039	166.0	134.5	108.3	94.8	82.6	73.5	68.1
	15 August 2020	279	15,318	279.0	222.5	182.7	151.0	131.6	115.3	102.9
	16 August 2020	197	15,515	197.0	238.0	214.0	186.3	160.2	142.5	127.0
Group 4	16 August 2020	320	18,265	320.0	300.0	288.7	315.8	319.0	319.8	315.3
	25 August 2020	441	18,706	441.0	380.5	347.0	326.8	340.8	339.3	337.1
	26 August 2020	371	19,077	371.0	406.0	377.3	353.0	335.6	345.8	343.9
	27 August 2020	323	19,400	323.0	347.0	378.3	363.8	347.0	333.5	342.6
	28 August 2020	299	19,699	299.0	311.0	331.0	358.5	350.8	339.0	328.6
	29 August 2020	248	19,947	248.0	273.5	290.0	310.3	336.4	333.7	326.0
Group 5	20 October 2020	91	25,424	91.0	74.5	75.0	79.0	77.8	72.7	78.0
	21 October 2020	119	25,543	119.0	105.0	89.3	86.0	87.0	84.7	79.3
	22 October 2020	155	25,698	155.0	137.0	121.7	105.8	99.8	98.3	94.7
	23 October 2020	77	25,775	77.0	116.0	117.0	110.5	100.0	96.0	95.3
	24 October 2020	61	25,836	61.0	69.0	97.7	103.0	100.6	93.5	91.0
	25 October 2020	119	25,955	119.0	90.0	85.7	103.0	106.2	103.7	97.1
	26 October 2020	88	26,043	88.0	103.5	89.3	86.3	100.0	103.2	101.4
	27 October 2020	103	26,146	103.0	95.5	103.3	92.8	89.6	100.5	103.1

**Table 2:** Groups and cases by period for forecast analysis.

Group	Case	Date	Group	Case	Date
1	Case 1	20 January 2020~28 February 2020	4	Case 11	20 January 2020~26 August 2020
	Case 2	20 January 2020~29 February 2020		Case 12	20 January 2020~27 August 2020
	Case 3	20 January 2020~2 March 2020		Case 13	20 January 2020~28 August 2020
	Case 4	20 January 2020~2 March 2020		Case 14	20 January 2020~29 August 2020
2	Case 5	20 January 2020~5 May 2020	5	Case 15	20 January 2020~21 October 2020
	Case 6	20 January 2020~6 May 2020		Case 16	20 January 2020~22 October 2020
	Case 7	20 January 2020~7 May 2020		Case 17	20 January 2020~23 October 2020
3	Case 8	20 January 2020~13 August 2020		Case 18	20 January 2020~25 October 2020
	Case 9	20 January 2020~14 August 2020		Case 19	20 January 2020~26 October 2020
	Case 10	20 January 2020~15 August 2020	Recent Case		20 January 2020~27 October 2020

**Table 3:** Results of auto regressive integrated moving average (ARIMA) models for Group 1 (Case 1–4).

Case	Model	RMSE	MAPE	MAE
1	ARIMA (5,2,5)	41.181	279.699	21.819
	ARIMA (5,2,3)	43.399	248.562	24.118
	ARIMA (5,2,4)	43.842	245.299	23.871
	ARIMA (5,2,2)	43.898	210.373	23.854
	ARIMA (3,2,3)	44.380	170.642	22.634
	ARIMA (4,2,2)	44.985	186.538	25.879
2	ARIMA (5,2,5)	41.618	280.246	22.416
	ARIMA (5,2,4)	42.445	264.567	23.586
	ARIMA (4,2,5)	43.134	185.019	23.488
	ARIMA (5,2,3)	43.212	233.869	24.876
	ARIMA (4,2,4)	43.358	197.110	25.585
	ARIMA (4,2,2)	44.134	180.253	25.783
3	ARIMA (5,2,5)	49.641	162.569	25.548
	ARIMA (5,2,4)	53.291	185.799	28.713
	ARIMA (5,2,3)	53.474	185.343	29.178
	ARIMA (4,2,3)	53.571	197.765	31.706
	ARIMA (4,2,4)	54.185	196.412	31.613
	ARIMA (5,2,1)	56.478	200.521	33.533
4	ARIMA (5,2,5)	49.869	149.950	25.762
	ARIMA (5,2,2)	52.173	177.074	29.535
	ARIMA (5,2,4)	52.491	172.811	28.209
	ARIMA (5,2,3)	52.593	177.511	28.595
	ARIMA (4,2,4)	53.320	191.480	30.849
	ARIMA (4,2,3)	53.340	184.271	30.593

**Table 4:** Results of ARIMA models for Group 2 (case 5–7).

Case	Model	RMSE	MAPE	MAE
5	ARIMA (2,2,5)	56.172	5.963	27.920
	ARIMA (5,2,5)	56.428	5.968	27.800
	ARIMA (3,2,5)	56.471	5.885	28.122
	ARIMA (4,2,5)	56.711	5.965	28.064
	ARIMA (5,2,3)	56.901	5.805	28.879
	ARIMA (1,2,5)	57.573	5.741	29.995
6	ARIMA (2,2,5)	55.895	5.719	27.720
	ARIMA (5,2,5)	56.147	5.748	27.637
	ARIMA (3,2,5)	56.185	5.668	27.886
	ARIMA (4,2,5)	56.424	5.708	27.934
	ARIMA (5,2,3)	56.589	5.737	28.651
	ARIMA (1,2,5)	57.283	5.719	29.760
7	ARIMA (2,2,5)	55.629	5.656	27.576
	ARIMA (5,2,5)	55.856	5.705	27.451
	ARIMA (3,2,5)	55.911	5.697	27.726
	ARIMA (4,2,5)	56.074	5.880	27.739
	ARIMA (5,2,3)	56.314	5.772	28.552
	ARIMA (1,2,5)	57.000	5.821	29.559

**Table 5:** Results of ARIMA models for Group 3 (case 8–10).

Case	Model	RMSE	MAPE	MAE
8	ARIMA (2,2,5)	41.712	3.338	21.422
	ARIMA (3,2,5)	41.842	3.324	21.452
	ARIMA (5,2,4)	41.908	3.357	21.539
	ARIMA (5,2,3)	42.149	3.332	21.863
	ARIMA (1,2,5)	42.659	3.443	22.016
	ARIMA (4,2,5)	43.019	3.400	21.602
9	ARIMA (2,2,5)	41.912	3.826	21.639
	ARIMA (3,2,5)	41.948	3.929	21.489
	ARIMA (5,2,3)	42.327	3.750	21.909
	ARIMA (5,2,4)	42.670	3.789	22.007
	ARIMA (1,2,5)	42.830	3.941	22.299
	ARIMA (4,2,5)	43.182	3.658	21.863
10	ARIMA (2,2,5)	42.796	5.012	22.084
	ARIMA (4,2,5)	42.953	4.557	22.098
	ARIMA (5,2,4)	42.961	4.570	22.007
	ARIMA (5,2,3)	43.140	5.146	22.399
	ARIMA (1,2,5)	43.640	5.178	22.863
	ARIMA (2,2,3)	44.011	4.337	22.637

**Table 6:** Results of ARIMA models for Group 4 (case 11–14).

Case	Model	RMSE	MAPE	MAE
11	ARIMA (5,2,5)	44.253	5.333	23.426
	ARIMA (3,2,5)	44.313	5.304	23.198
	ARIMA (4,2,5)	44.417	5.334	23.291
	ARIMA (2,2,5)	44.558	5.417	23.484
	ARIMA (5,2,3)	44.904	5.330	23.665
	ARIMA (5,2,4)	45.051	5.390	23.746
12	ARIMA (3,2,5)	44.405	4.711	23.263
	ARIMA (4,2,5)	44.476	4.797	23.207
	ARIMA (2,2,5)	44.643	4.866	23.504
	ARIMA (5,2,3)	45.015	4.755	23.653
	ARIMA (5,2,4)	45.314	4.626	23.801
	ARIMA (1,2,5)	45.397	4.467	23.848
13	ARIMA (3,2,5)	44.471	4.190	23.417
	ARIMA (4,2,5)	44.536	4.313	23.344
	ARIMA (2,2,5)	44.675	4.372	23.506
	ARIMA (5,2,4)	44.779	4.452	23.463
	ARIMA (5,2,5)	44.798	4.352	23.527
	ARIMA (4,2,4)	44.867	4.352	23.050
14	ARIMA (3,2,5)	44.449	3.845	23.491
	ARIMA (4,2,5)	44.484	4.056	23.391
	ARIMA (5,2,4)	44.707	4.247	23.410
	ARIMA (5,2,5)	44.714	4.188	23.457
	ARIMA (4,2,4)	44.773	4.242	22.997
	ARIMA (5,2,3)	44.941	4.116	23.740

**Table 7:** Results of ARIMA models for Group 5 (case 15–19).

Case	Model	RMSE	MAPE	MAE
15	ARIMA (3,2,5)	41.744	2.511	23.107
	ARIMA (4,2,5)	41.811	2.513	23.150
	ARIMA (2,2,5)	41.892	2.495	23.300
	ARIMA (5,2,3)	42.234	2.524	23.307
	ARIMA (5,2,4)	42.308	2.536	23.433
	ARIMA (1,2,5)	42.622	2.509	23.948
16	ARIMA (4,2,5)	41.852	2.647	23.268
	ARIMA (2,2,5)	41.932	2.624	23.408
	ARIMA (5,2,3)	42.285	2.636	23.714
	ARIMA (5,2,4)	42.319	2.651	23.534
	ARIMA (1,2,5)	42.631	2.628	24.053
	ARIMA (4,2,4)	42.779	2.609	23.730
17	ARIMA (3,2,5)	41.957	2.429	23.375
	ARIMA (4,2,5)	42.030	2.428	23.410
	ARIMA (2,2,5)	42.100	2.419	23.532
	ARIMA (5,2,3)	42.416	2.432	23.727
	ARIMA (1,2,5)	42.782	2.414	24.175
	ARIMA (4,2,4)	42.892	2.458	23.869
18	ARIMA (3,2,5)	41.938	2.444	23.457
	ARIMA (2,2,5)	42.072	2.449	23.619
	ARIMA (5,2,4)	42.349	2.489	23.824
	ARIMA (5,2,3)	42.366	2.472	23.791
	ARIMA (5,2,5)	42.390	2.498	23.826
	ARIMA (1,2,5)	42.714	2.447	24.212
19	ARIMA (3,2,5)	41.871	2.392	23.431
	ARIMA (4,2,5)	41.940	2.391	23.491
	ARIMA (2,2,5)	42.005	2.397	23.589
	ARIMA (5,2,2)	42.278	2.386	23.846
	ARIMA (5,2,3)	42.319	2.398	23.796
	ARIMA (5,2,5)	42.401	2.394	23.943

**Table 8:** Results of ARIMA models for each group and case based on SSE.

Group	Case	Model	SSE	Rank of SSE	Note
1	2	ARIMA (4,2,5)	138,245,907	1	∆t=2,3,4
	4	ARIMA (5,2,5)	159,104,779	2	∆t=6,7
	3	ARIMA (5,2,5)	195,270,591	3	∆t=4,5
	3	ARIMA (5,2,4)	273,033,961	4	∆t=4,5
	4	ARIMA (5,2,4)	311,756,668	5	∆t=6,7
2	7	ARIMA (5,2,5)	21,750	1	∆t=5
	5	ARIMA (5,2,5)	978,159	2	∆t=1,2,7
	6	ARIMA (5,2,5)	182,580,231	3	∆t=3,4,6,7
	6	ARIMA (1,2,5)	250,929,996	4	∆t=3,4,6,7
	5	ARIMA (4,2,5)	282,621,031	5	∆t=1,2,7
3	10	ARIMA (1,2,5)	16,973,894	1	∆t=4,5,6,7
	9	ARIMA (4,2,5)	28,752,738	2	∆t=2,3
	9	ARIMA (5,2,3)	311,216,609	3	∆t=2,3
	8	ARIMA (5,2,3)	360,558,068	4	∆t=1
	8	ARIMA (5,2,4)	948,734,643	5	∆t=1
4	14	ARIMA (4,2,5)	26,281,173	1	∆t=5
	14	ARIMA (5,2,3)	30,701,687	2	∆t=5
	12	ARIMA (5,2,3)	39,839,429	3	∆t=2,6,7
	12	ARIMA (4,2,5)	43,645,283	4	∆t=2,6,7
	11	ARIMA (5,2,3)	47,148,618	5	∆t=1
5	17	ARIMA (2,2,5)	45,812	1	∆t=5
	18	ARIMA (3,2,5)	48,181	2	∆t=6
	19	ARIMA (3,2,5)	64,905	3	∆t=7
	15	ARIMA (1,2,5)	397,393	4	∆t=1,2
	19	ARIMA (2,2,5)	2,161,447	5	∆t=7

**Table 9:** Criteria of confirmed cases according to ARIMA.

Model	RMSE	MAPE	MAE
ARIMA (3,2,5)	53.031	4.190	29.780
ARIMA (5,2,4)	53.323	4.216	29.925
ARIMA (2,2,5)	53.333	4.449	30.232
ARIMA (5,2,3)	53.591	4.120	30.061
ARIMA (4,2,3)	54.150	4.914	30.567
ARIMA (4,2,4)	54.177	4.811	30.602
ARIMA (5,2,5)	54.638	4.296	30.976
ARIMA (1,2,5)	54.680	3.860	30.569
ARIMA (3,2,4)	55.385	4.568	31.593
ARIMA (0,2,5)	55.621	4.609	30.879

**Table 10:** Prediction of cumulative confirmed cases according to the best models with 95% confidence interval.

Date	Real Data	Based on RMSE and MAE ARIMA (3,2,5)			Based on MAPE ARIMA (1,2,5)		
		Forecast	UCL	LCL	Forecast	UCL	LCL
28 December 2020	58,714	58,532	58,636	58,427	58,533	58,640	58,425
29 December 2020	59,764	59,456	59,668	59,243	59,477	59,697	59,256
30 December 2020	60,731	60,428	60,756	60,101	60,417	60,755	60,079
31 December 2020	61,758	61,448	61,912	60,984	61,358	61,832	60,883
1 January 2021	62,578	62,432	63,046	61,818	62,248	62,875	61,622
2 January 2021	63,235	63,327	64,106	62,547	63,113	63,920	62,306
3 January 2021	64,255	64,153	65,125	63,180	63,964	64,983	62,945
4 January 2021	64,969	64,975	66,175	63,775	64,809	66,070	63,548
5 January 2021	65,807	65,847	67,308	64,386	65,651	67,181	64,121
6 January 2021	66,676	66,770	68,515	65,025	66,493	68,318	64,669
7 January 2021	67,350	67,710	69,752	65,667	67,336	69,477	65,195
8 January 2021	67,991	68,628	70,978	66,278	68,181	70,660	65,702
9 January 2021	68,648	69,515	72,185	66,845	69,028	71,864	66,192
10 January 2021	69,099	70,389	73,394	67,384	69,877	73,088	66,665

## Data Availability

Data available in a publicly accessible repository.

## References

[1] World Economic Outlook: A Long and Difficult Ascent. https://www.imf.org/en/Publications/WEO/Issues/2020/09/30/world-economic-outlook-october-2020.

[2] Maliszewska M., Mattoo A., van der Mensbrugghe D. (2020). The Potential Impact of COVID-19 on GDP and Trade: A Preliminary Assessment. World Bank Policy Res. Work. Paper.

[3] WHO Director-General’s Opening Remarks at the Media Briefing on COVID-19—11 March 2020. https://www.who.int/director-general/speeches/detail/who-director-general-s-opening-remarks-at-the-media-briefing-on-covid-19---11-march-2020.

[4] Past Pandemics. https://www.cdc.gov/flu/pandemic-resources/basics/past-pandemics.html.

[5] Johns Hopkins CSSE ‘COVID19 Daily Reports’. https://www.arcgis.com/apps/opsdashboard/index.html#/bda7594740fd40299423467b48e9ecf6.

[6] Guan P., Huang D.S., Zhou B. (2004). Sen Forecasting model for the incidence of hepatitis A based on artificial neural network. World J. Gastroenterol..

[7] Earnest A., Chen M.I., Ng D., Leo Y.S. (2005). Using autoregressive integrated moving average (ARIMA) models to predict and monitor the number of beds occupied during a SARS outbreak in a tertiary hospital in Singapore. BMC Health Serv. Res..

[8] Liu Q., Liu X., Jiang B., Yang W. (2011). Forecasting incidence of hemorrhagic fever with renal syndrome in China using ARIMA model. BMC Infect. Dis..

[9] Wu W., Guo J., An S., Guan P., Ren Y., Xia L., Zhou B. (2015). Comparison of two hybrid models for forecasting the incidence of hemorrhagic fever with renal syndrome in Jiangsu Province, China. PLoS ONE.

[10] Nsoesie E.O., Beckman R.J., Shashaani S., Nagaraj K.S., Marathe M.V. (2013). A Simulation Optimization Approach to Epidemic Forecasting. PLoS ONE.

[11] Chen Y., Leng K., Lu Y., Wen L., Qi Y., Gao W., Chen H., Bai L., An X., Sun B. (2020). Epidemiological features and time-series analysis of influenza incidence in urban and rural areas of Shenyang, China, 2010-2018. Epidemiol. Infect..

[12] Webb G., Magal P., Liu Z., Seydi O. (2020). A model to predict COVID-19 epidemics with applications to South Korea, Italy, and Spain. SIAM News.

[13] Alakus T.B., Turkoglu I. (2020). Comparison of deep learning approaches to predict COVID-19 infection. Chaos Solitons Fractals.

[14] Pham H. (2020). On estimating the number of deaths related to Covid-19. Mathematics.

[15] Pham H. (2020). Predictive modeling on the number of Covid-19 death toll in the united states considering the effects of coronavirus-related changes and Covid-19 recovered cases. Int. J. Math. Eng. Manage. Sci..

[16] Pham H. (2020). Estimating the COVID-19 death toll by considering the time-dependent effects of various pandemic restrictions. Mathematics.

[17] Arias V., Alberto M. (2020). Using generalized logistics regression to forecast population infected by Covid-19. arXiv.

[18] Kumar P., Singh R.K., Nanda C., Kalita H., Patairiya S., Sharma Y.D., Rani M., Bhagavathula A.S. (2020). Forecasting COVID-19 impact in India using pandemic waves Nonlinear Growth Models. MedRxiv.

[19] Petropoulos F., Makridakis S., Stylianou N. (2020). COVID-19: Forecasting confirmed cases and deaths with a simple time-series model. Int. J. Forecast..

[20] Ceylan Z. (2020). Estimation of COVID-19 prevalence in Italy, Spain, and France. Sci. Total Environ..

[21] Alzahrani S.I., Aljamaan I.A., Al-Fakih E.A. (2020). Forecasting the spread of the COVID-19 pandemic in Saudi Arabia using ARIMA prediction model under current public health interventions. J. Infect. Public Health.

[22] Yang Q., Wang J., Ma H., Wang X. (2020). Research on COVID-19 based on ARIMA modelΔ—Taking Hubei, China as an example to see the epidemic in Italy. J. Infect. Public Health.

[23] Kufel T. (2020). ARIMA-based forecasting of the dynamics of confirmed Covid-19 cases for selected European countries. Equilibrium. Q. J. Econ. Econ. Policy.

[24] Benvenuto D., Giovanetti M., Vassallo L., Angeletti S., Ciccozzi M. (2020). Application of the ARIMA model on the COVID-2019 epidemic dataset. Data Br..

[25] Liu Z., Magal P., Webb G. (2020). Predicting the number of reported and unreported cases for the COVID-19 epidemics in China, South Korea, Italy, France, Germany and United Kingdom. J. Theor. Biol..

[26] Yang S., Cao P., Du P., Wu Z., Zhuang Z., Yang L., Yu X., Zhou Q., Feng X., Wang X. (2020). Early estimation of the case fatality rate of COVID-19 in mainland China: A data-driven analysis. Ann. Transl. Med..

[27] Payne J.L., Morgan A. (2020). COVID-19 and Violent Crime: A comparison of recorded offence rates and dynamic forecasts (ARIMA) for March 2020 in Queensland, Australia. Preprint.

[28] Matthew E., Adeyinka O. (2020). Application of Hierarchical Polynomial Regression Models to Predict Transmission of COVID-19 at Global Level. Int. J. Clin. Biostat. Biom..

[29] Ilie O.D., Cojocariu R.O., Ciobica A., Timofte S.I., Mavroudis I., Doroftei B. (2020). Forecasting the spreading of COVID-19 across nine countries from Europe, Asia, and the American continents using the arima models. Microorganisms.

[30] Song J.-Y., Yun J.-G., Noh J.-Y., Cheong H.-J., Kim W.-J. (2020). Covid-19 in South Korea—Challenges of Subclinical Manifestations. N. Engl. J. Med..

[31] Cases in Korea. http://ncov.mohw.go.kr/en/bdBoardList.do?brdId=16&brdGubun=161&dataGubun=&ncvContSeq=&contSeq=&board_id=.

[32] Protestant Churches under Fire for Holding Sunday Services Despite Coronavirus Epidemic. http://news.koreaherald.com/view.php?ud=20200317000794&ACE_SEARCH=1.

[33] Korea Reports 323 New COVID-19 Cases. http://news.koreaherald.com/view.php?ud=20200829000051&ACE_SEARCH=1.

[34] COVID-19 Cases See Largest Daily Increase since August. Ttp://news.koreaherald.com/view.php?ud=20201125000190&ACE_SEARCH=1.

[35] Box G.E., Jenkins G.M., Reinsel G.C., Ljung G.M. (2015). Time Series Analysis: Forecasting and Control.

